# Protein interaction potential landscapes for yeast replicative aging

**DOI:** 10.1038/s41598-021-86415-8

**Published:** 2021-03-30

**Authors:** Hao-Bo Guo, Mehran Ghafari, Weiwei Dang, Hong Qin

**Affiliations:** 1 Department of Computer Science and Engineering, The University of Tennessee at Chattanooga, Chattanooga, TN 37405 USA; 2 SimCenter, The University of Tennessee at Chattanooga, Chattanooga, TN 37405 USA; 3 Department of Biology, Geology and Environmental Science, The University of Tennessee at Chattanooga, Chattanooga, TN 37405 USA; 4 Huffington Center on Aging, Baylor College of Medicine, Houston, TX 77030 USA; 5 Present Address: Materials and Manufacturing Directorate, Air Force Research Laboratory, Wright-Patterson AFB, Dayton, OH 45433 USA

**Keywords:** Systems biology, Computer modelling, Dynamic networks, Genetic interaction, Time series

## Abstract

We proposed a novel interaction potential landscape approach to map the systems-level profile changes of gene networks during replicative aging in *Saccharomyces cerevisiae*. This approach enabled us to apply quasi-potentials, the negative logarithm of the probabilities, to calibrate the elevation of the interaction landscapes with young cells as a reference state. Our approach detected opposite landscape changes based on protein abundances from transcript levels, especially for intra-essential gene interactions. We showed that essential proteins play different roles from hub proteins on the age-dependent interaction potential landscapes. We verified that hub proteins tend to avoid other hub proteins, but essential proteins prefer to interact with other essential proteins. Overall, we showed that the interaction potential landscape is promising for inferring network profile change during aging and that the essential hub proteins may play an important role in the uncoupling between protein and transcript levels during replicative aging.

## Introduction

Studies of replicative lifespan (RLS) in budding yeast, *Saccharomyces cerevisiae*, provided mechanistic insights on cellular aging^1^, and contributed to the understanding of aging in other organisms including humans^2–5^. Deletions of single genes, such as *FOB1*^6^, and suppression or overexpression of genes, such as *SIR2*^7,8^, could change the yeast RLS. Other factors that affect lifespan, such as caloric restriction (CR), were also found dependent on the genotypes^9^. A recent report found overexpression of both *SIR2* and *HAP4* genes leads to a cell state with considerably long RLS^10^.

Gene network is critical to understand the pleiotropic nature of aging^11–13^. It was shown that cellular aging can be viewed as an emergent phenomenon of a gene interaction network with stochastic loss of interactions to essential proteins^14^. A recent study showed that the uncoupling between protein and transcript levels is strongly connected to yeast aging^15^. The time-series transcriptome and proteome data of this work also involved extensive numbers of essential proteins and genes^15^.

The pleiotropic nature of yeast aging is also reflected at the transcriptome and proteome levels. Transcriptional fidelity is associated with aging and longevity in yeast^11,12^. The translational efficiencies of different proteins vary up to hundreds of folds in yeast, however, protein and mRNA levels were highly correlated^13^. Increased noises in both transcriptome and proteome during aging could be part of the cascading events leading to replicative aging and eventual cell death.

The protein interaction network (PIN) offers a backbone for cellular functions. Proteins with similar functions tend to interact with each other and form functional modules^14^. The PIN is often modeled as a graph composed of proteins as vertices and interactions as edges^16–18^. The protein–protein interactions (PPIs) are important factors in the genotype-to-phenotype relationships^19^. It was proposed that aging preferentially attacks key regulatory nodes important for the stability of the PIN^20^. Age-specific PIN was found to suggest key players in aging^21^.

The PPI must bear a weight (strength) depending on both biophysical properties and spatial/temporal distributions of the proteins. In the crowded environment of the cell a PPI is also the subject of competitions and/or inferences by other molecules including the solvents. This situation complicates the PPI detections and, in many cases, only the “binary” PINs—i.e., “1” for interaction and “0” for no interaction—can be estimated, such as those reported in the BioGRID^22^. Nevertheless, we propose in this work that a “quasi-potential” approach could be utilized to weight the PPIs using the age-dependent transcriptome and proteome dataset of the yeast *S. cerevisiae*^15^ and corresponding PIN^22^. We constructed the Protein Interaction Potential landscapes (PIPLs) based on the age-dependent potentials. We observed opposite landscape changes from protein levels and those based on transcript levels. The results also suggest that interactions to essential hub proteins may play an important role in the uncoupling of proteome and transcriptome during aging.

## Result

We hypothesize that the uncoupling of proteome and transcriptome in replicative aging may lead to protein–protein interaction profile changes, which motivated us to construct age-dependent PIPLs using the age-dependent proteome and transcriptome data along the yeast replicative aging^15^.

### Interaction potential landscape on lifespans suggest roles of essential genes for uncoupling of protein and transcript levels during aging

**Figure 1:**
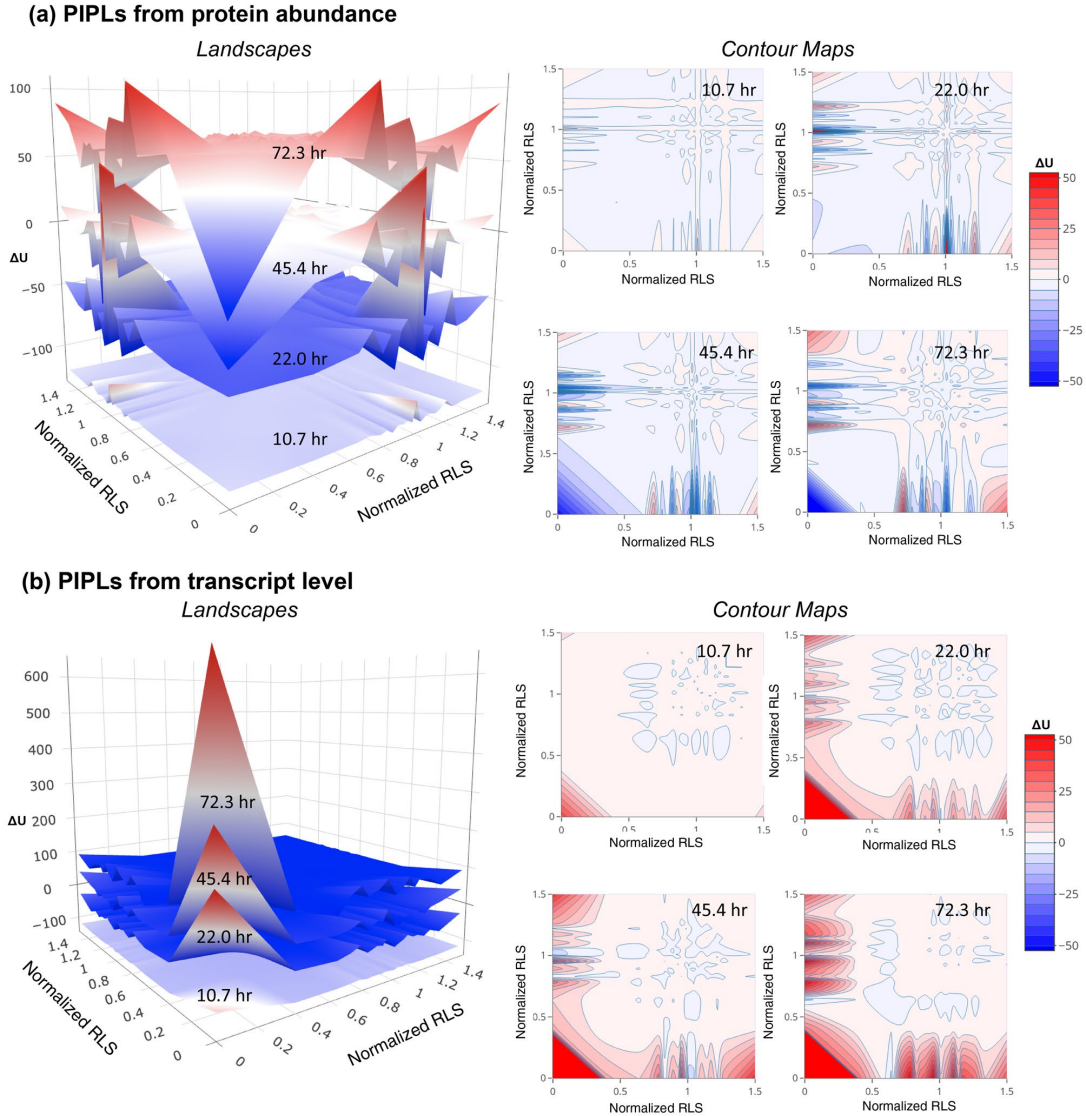
Opposite trends of intra-essential protein interactions during replicative aging as shown by the age-dependent PIPLs on the dimension of normalized RLSs. (**a**) Elevations estimated by proteome on landscapes (left) and corresponding contours (right). (**b**) Elevations estimated by transcriptome on landscapes (left) and corresponding contours (right). The relative quasi potentials (ΔU) were calculated using the youngest cells at 7.8 h as the reference ground state. The horizontal axes of the landscapes are the RLSs of the single-gene mutants normalized by the WT measurement at the same conditions (normalized RLSs). The essential genes have normalized RLSs of 0. The landscape based on protein abundance shows that the essential-vs-essential interaction region gradually fell into a low-potential basin. However, in the landscape based on gene expression levels the essential-vs-essential interaction region gradually rose to a high-potential ridge. The landscapes at different ages have been vertically shifted for a better visualization. The color bar shows the relative quasi potentials in the contour maps. A relative color scheme (blue for low and red for high) is applied in landscapes.

To gauge changes in the interaction profiles during replicative aging, we constructed the age-dependent PIPLs on the dimension of RLS. We used RLS ratios between the RLS of single-gene deletion mutants and those of the wild type cells cultivated under the same YPD (2% glucose) conditions, which is referred to as the normalized RLS in present work. The normalized RLS of essential proteins (genes) is 0, as the cell will not be viable after the deletion of a single essential gene. The probabilistic nature of the landscape enabled us to apply quasi-potentials to quantify the elevations of the landscape. We choose the young cells collected at the age of 7.8 h as the ground state (Fig. 1), a term borrowed from the embryonic stem cell studies^23^. Note that the quasi-potential used for the landscapes was calculated as the negative logarithm of the interaction probability based on the law of mass action (“Methods and materials”). Therefore, as compared with the ground state, the ridges on the landscape indicate that the interacting pairs need to acquire “high potential”, whereas basins represent that the interacting pairs possess “low potential” for interactions to occur.

To estimate the changing elevations on landscapes during aging, we used two approaches: One based on the proteome (Fig. 1a) and one based on the transcriptome (Fig. 1b). We observed some strikingly opposite trends in the two landscapes for intra-essential interactions, especially at old ages. On the PIPL estimated from proteome, we found that intra-essential interactions form basins in old ages, corresponding to lower interaction potentials (higher probabilities) than the ground state young cells. On the PIPL estimated from transcriptome, we found that intra-essential interactions form peaks (higher interaction potentials or lower probabilities) in old ages. The PIPLs at selected age had been plotted, and we also visualized this opposite trend in corresponding two-dimensional contour maps (Fig. 1).

### Interaction potential landscape examined by node degrees and normalized RLS

**Figure 2:**
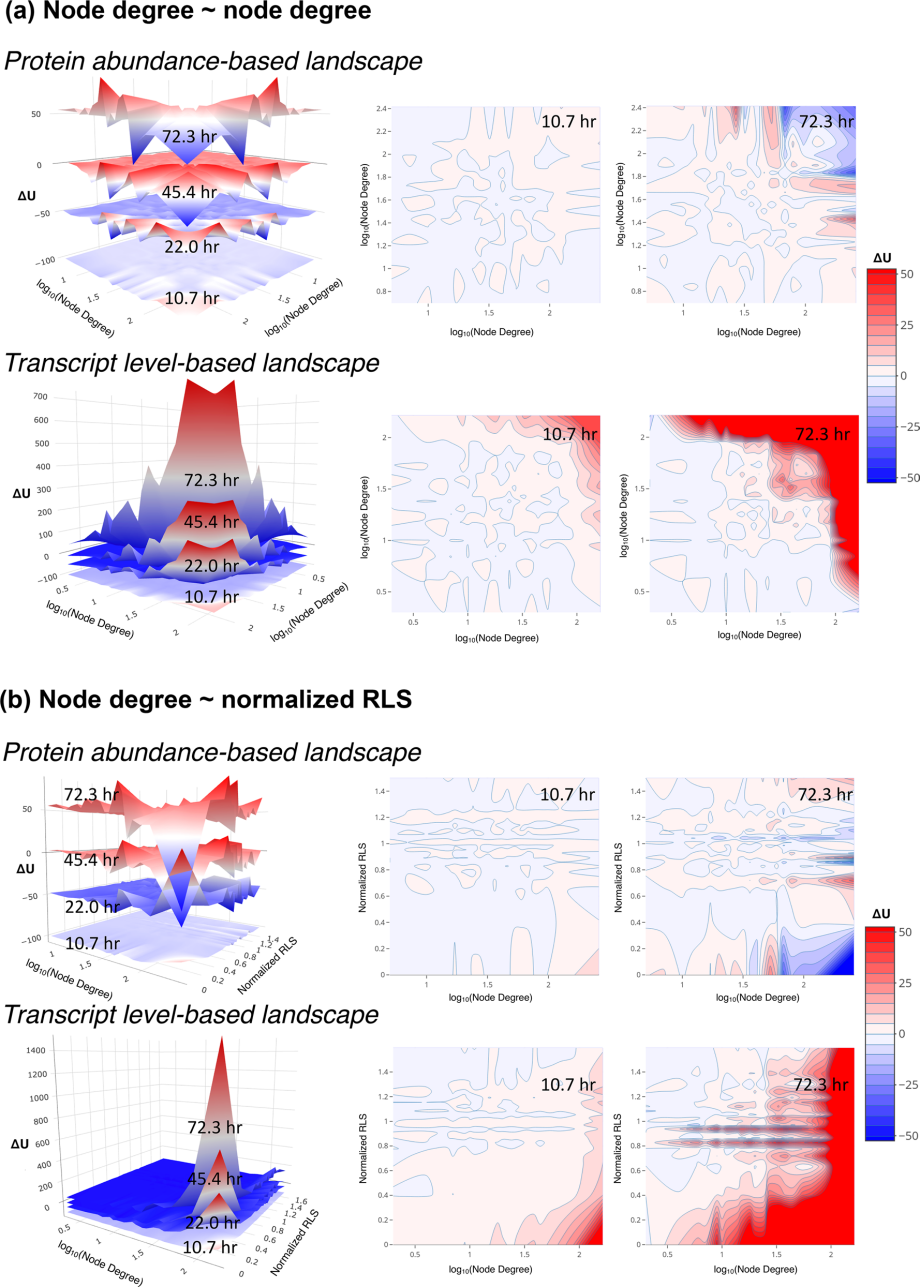
Dissimilarity between the essential and hub proteins from the PIPLs on dimensions of node degrees and normalized RLSs. (**a**) PIPLs on node degrees versus node degrees, and (**b**) PIPLs on node degrees versus normalized RLSs. The top panels show the PIPLs from protein abundances, and the bottom panels show the PIPLs from transcript levels. The contour maps of the aging cells at young (10.7 h) and old (72.3 h) are shown for comparisons. The landscapes at different ages have been vertically shifted for better visualization. The color bar shows the relative quasi-potentials in the contour maps. A relative color scheme (blue for low and red for high) is applied in landscapes.

To better understand the role of essential genes during aging, we examined the interaction potential landscapes by node degrees (Fig. 2a), because it was suggested that essential genes were often highly connected hubs in the protein networks^24^. Elevations of the landscape were estimated again with both protein and transcript levels.

Based on protein abundances, in the old cells at 72.3 h, the hub-versus-hub protein interactions also fell into a basin (ΔU = –31) with the quasi-potential weaker than the essential-versus-essential protein interactions (ΔU = –83) shown in Fig. 1. The relative quasi-potentials for younger cells were even positive for younger cells from 10.7 to 22 h (ΔU = 0.5 to 1.8). The extents of the quasi-potentials at other regions were also smaller than those in the PIPL based on normalized RLSs in Fig. 1. For the PIPL based on transcript levels, the interactions with hub proteins, both from other hub and non-hub proteins, generally rose to ridges of the landscape.

We further examined the PIPL on both the node degrees and normalized RLSs (Fig. 2b). The interactions between the hub and essential proteins of the old cells at 72.3 h fell into a deep basin with low relative quasi-potential (ΔU = –94). However, those at younger ages from 10.7 to 26.8 h were located in ridges. Moreover, the hub-versus-essential interactions in the PIPL at 22.0 h were on a relatively high ridge of ΔU = 60. This analysis indicated that the hub proteins and essential proteins, though they are statistically correlated, may play different roles in aging.

### PIPL on dimensions of RLS versus protein turnover

**Figure 3:**
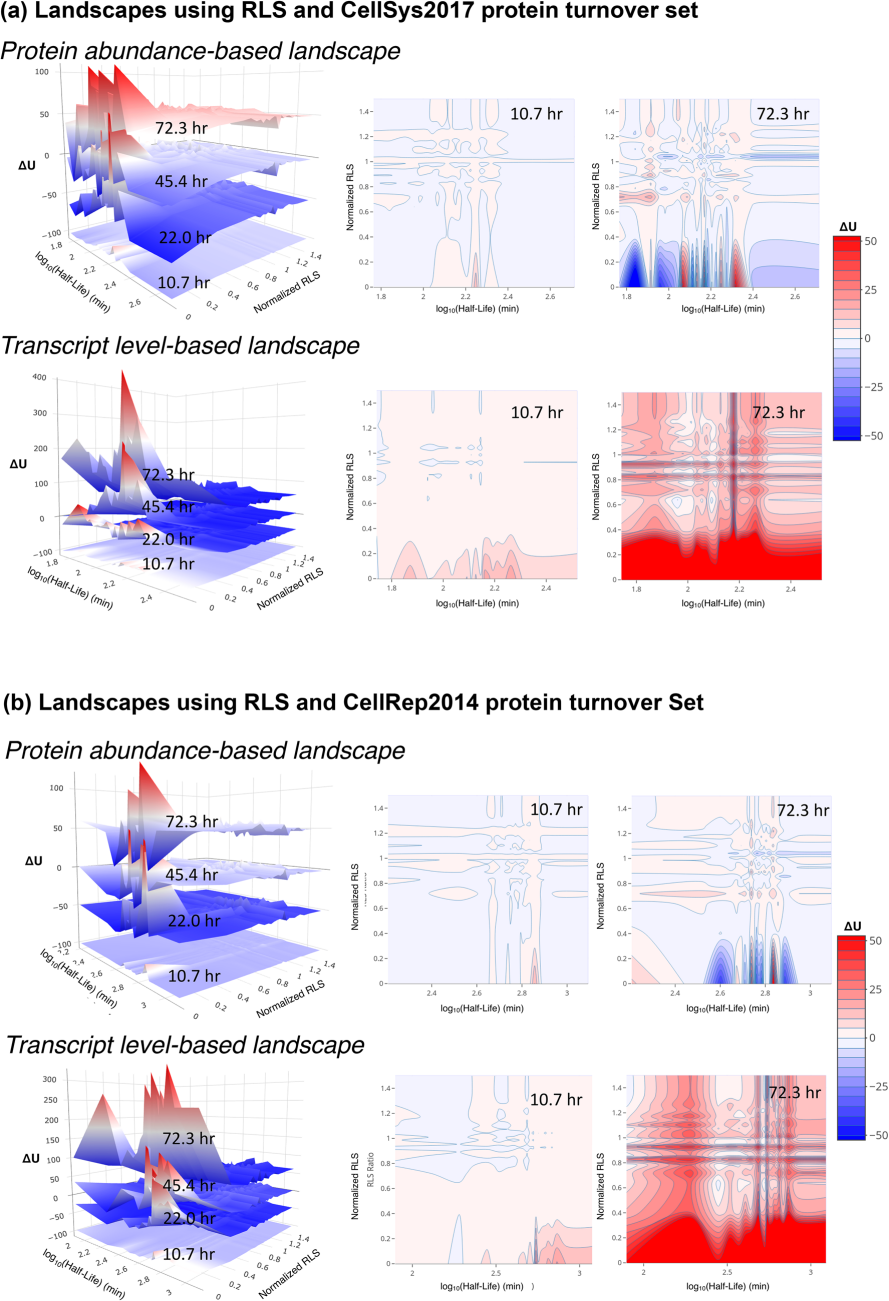
Interactions between essential proteins and short-lived proteins are noisy on the age-dependent PIPLs on dimensions of normalized RLSs and protein turnover rates. The protein turnover data were taken from (**a**) the CellSys2017 set and (**b**) the CellRep2014 set, respectively. The landscapes from both sets showed significant differences between the protein abundance-based (top panels) and transcript level-based (bottom panels) PIPLs. The contour maps of the landscapes of the aging cells at young (10.7 h) and old (72.3 h) are shown for comparisons. The landscapes at different ages have been vertically shifted for a better visualization. The color bar shows the relative quasi potentials in the contour maps. A relative color scheme (blue for low and red for high) is applied in landscapes.

The inversed trends between using the protein abundances and transcript levels may be associated with the protein aggregation during the aging process^25,26^. It may also be owing to the fact that many proteins in yeast have long half-lives^27^. A significant amount of proteins underwent non-exponential degradations in the cells and that the protein degradation rate seemed to be age-dependent^28^. The protein turnover rates were correlated protein functions and activities^29^, and the long-lived proteins may accumulate to high abundances in old cells even though their expression levels had been declined. Here, two independent protein turnover data sets, the CellSys2017 set^29^ and the CellRep2014 set^27^ had been used to construct the age-dependent PIPLs on the dimensions of normalized RLSs versus protein turnover rates (half-lives). Considerable differences can be found between the results of both sets (Fig. 3). However, they both interpreted that the short-turnover proteins, which may exhibit higher activities than the long-turnover proteins^29^, had larger fluctuations on the PIPLs of aging cells, especially in interactions with essential proteins. Note that log_10_-scales had been applied for the turnover rates, consistent with their distributions^27^.

Using the above landscapes, we noted that aging has a higher impact on the transcripts (mRNA) levels than to the protein abundances, with respect to the protein–protein interactions. For example, whereas based on the transcript levels for both turnover sets, the interactions with essential genes from all gene groups with different turnovers were in high potential, low probability states, in the protein abundance-based landscapes the interactions between certain groups of genes and the essential genes were dropped to low potential, high probability basins. Moreover, using the CellSys2017 set the low-turnover proteins also showed dropped quasi-potentials or increased interaction probabilities with the essential genes.

Based on the transcript levels (bottom panels), the landscapes using both turnover sets showed elevated quasi-potentials, especially at old age (Fig. 3). However, based on protein abundances (top panels), fluctuations of both elevations and declines in the quasi-potentials had been found, consistent with the uncoupling trend between protein and mRNA levels^15^. Overall, in all the landscapes we observed large fluctuations attributed to the essential genes (normalized RLS of 0) and their interactions with the low-turnover proteins. The interactions between essential genes and high-turnover proteins are relatively stable during aging, according to the protein-abundance-based PIPLs, but not in the gene-expression-based PIPLs.

### A protein interaction potential landscape based on network permutations

**Figure 4:**
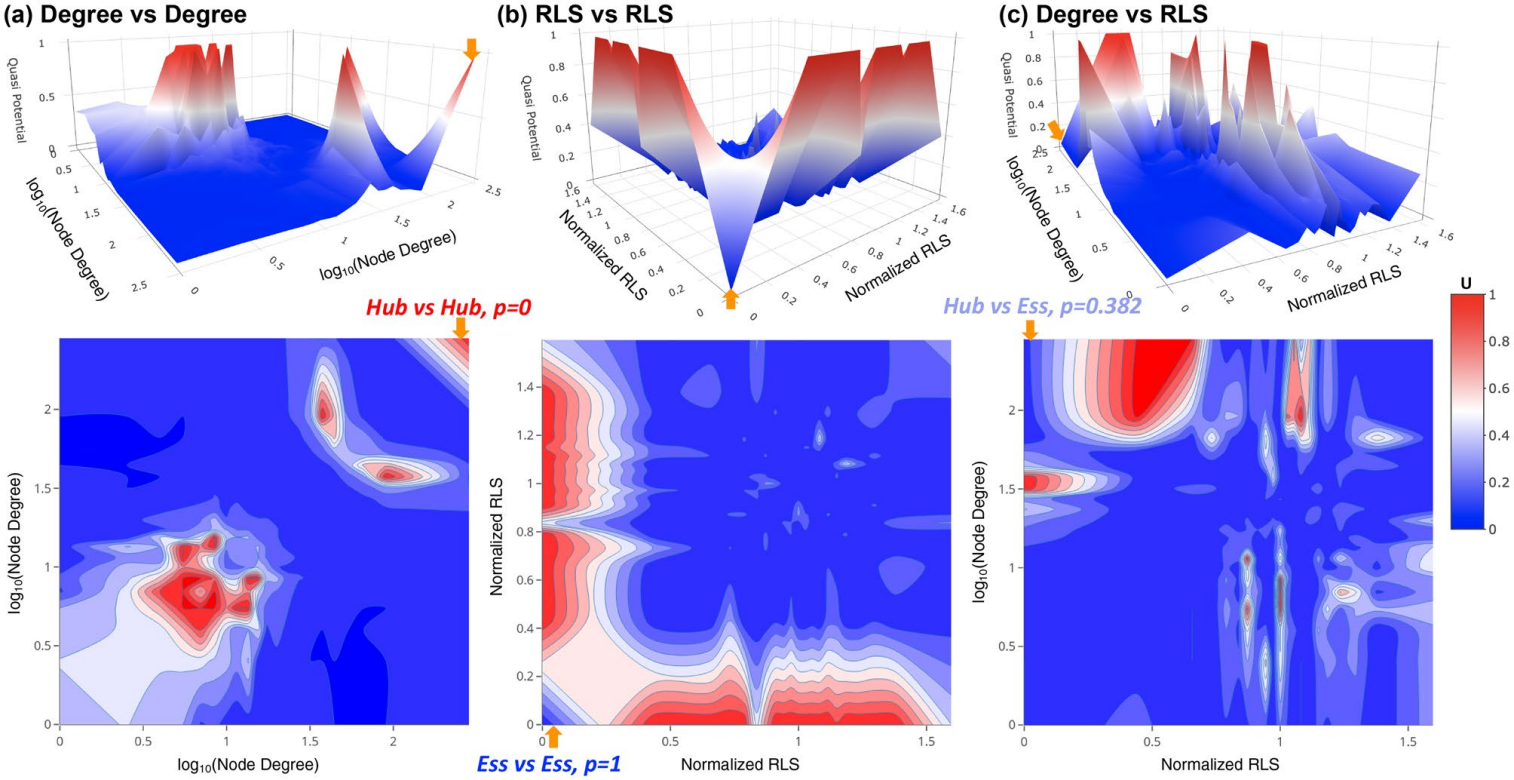
The proteome-wide protein interaction potential landscapes reveal differences between the hub and essential proteins. (**a**) The protein interaction landscape on space of the node degrees showed that the hub proteins avoid interactions with other hub proteins (*p* = 0). (**b**) The essential proteins favor interactions with other essential proteins (*p* = 1). (**c**) A moderate ratio of hub-essential interactions (*p* = 0.382) was observed. The hub-hub, essential-essential, and hub-essential interaction regions are highlighted by orange arrows on the landscapes and contour maps. The probabilities *p* indicates the ratio of null network models that showed fewer interactions than the empirical PIN: *p* = 1 (such as the essential-essential interactions) indicates the PIN has more interactions than all null models and *p* = 0 (such as the hub-hub interactions) indicates the PIN has fewer interactions than all null models, respectively. The quasi potential is calculated as U = − log*p*, the base of the logarithm function is chosen such that U is in range of [0,1]. 22,188 null network models have been used in this work and the *p* = 0 (i.e., *p* < 1/22,188) ratios were converted to *p* = 1/22,189 for estimation of the quasi potential, U. Top panels show the landscapes and the bottom panels show the 2D contour maps. The color bar for both the landscapes and contours are shown at right.

To further understand why lower relative quasi-potential (or higher probability) have been found in the essential-versus-essential interactions based on the proteomics, despite the transcript level trend predicted the opposite direction, we constructed another proteome-wide, global landscape model which calculates the probabilities of that the empirical PIN having more interactions than the random null models. As for the age-dependent landscape models, quasi-potentials were calculated as the negative logarithm of the probabilities to infer basins and hills on this landscape. Similar method had been previously applied to a smaller yeast PIN and observed that the high-degree, hub proteins tend to avoid interactions with the other hub proteins, which was suggested to contribute to the stability of the PIN^30^. This approach had been recently applied to estimate the association strengths between different categories of proteins using a human PIN^17^. Figure 4 shows the global landscape in the variable spaces of node degrees and RLS ratios (i.e., normalized RLS) of single-gene deletion mutations.

The network permutation-based landscape on the node-degree space showed that the hub proteins (high-degree proteins in the PIN) tend to avoid interactions with other hub proteins yet favor the interactions with non-hub proteins (Fig. 4a), in agreement with previous reports from a smaller yeast PIN^30^. Originally observed in yeast PIN, it had been proposed that the hub-proteins in the PIN tend to be essential proteins and vice versa^24^. This centrality-lethality rule hypothesis had been confirmed in the PINs of other organisms^31^ as well as the genetic interaction network (GIN) of yeast^32^. On the global landscape on the RLS space, however, the global landscape indicated that the essential proteins strongly favor the interactions with other essential proteins but tend to avoid the interactions with non-essential proteins (Fig. 4b). The probability of the interactions between essential proteins and hub proteins were moderate (Fig. 4c). Therefore, the essential proteins and hub proteins should not be treated equally in these landscapes. The above observation might be in conflict with the well-known centrality-lethality rule^24,31,33^.

It is worth noting that the essential genes did have higher degrees (average of 63.3) than non-essential genes (average of 32.7). Based on the landscape on the node degree-RLS space (Fig. 4c), we also observed that the interaction densities between hub proteins and essential proteins are significantly stronger than those between the hub proteins and nonessential proteins, also as shown in Figure S1 in the supplementary materials. However, despite a higher degree in the PIN, a hub protein is not necessarily essential. For example, the most-connected hub protein *DHH1* (YDL160C) has 3,605 interactions in the PIN but is nonessential and the Δ*DHH1* mutant has a normalized RLS of 0.825 compared to the wild type.

### Essential hub genes have increased protein abundances in aging cells

**Figure 5:**
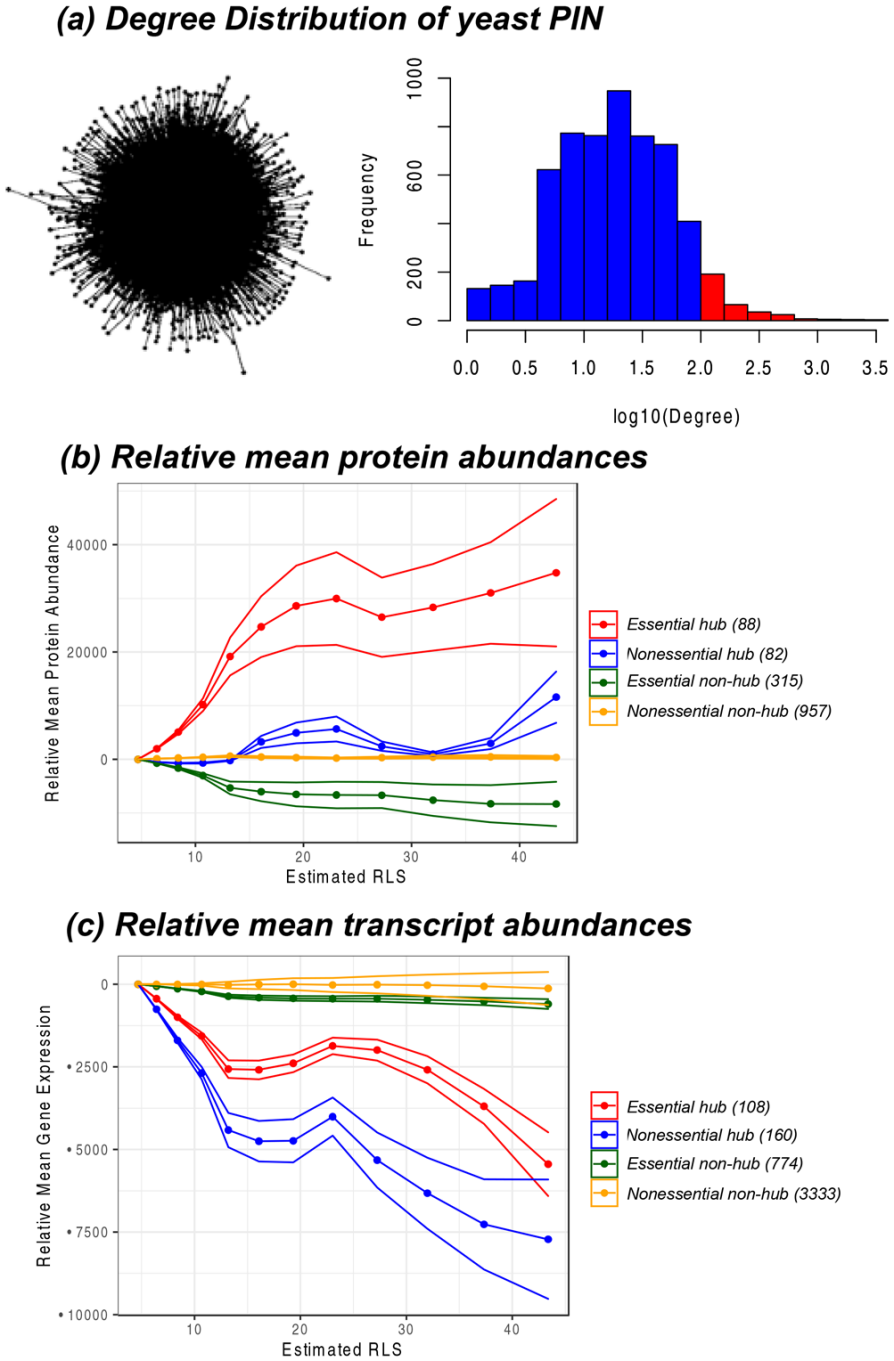
Essential hub proteins (or genes) may account for the opposite trends on the aging landscapes from proteomes and transcriptomes. All proteins (genes) were put in four categories including essential hub (red), nonessential hub (blue), essential non-hub (yellow), and nonessential non-hub proteins or genes. (**a**). Distribution of the node degrees in the yeast PIN. A snapshot of the PIN was shown at the left. The hub proteins (red bars) were selected as the node degree k ≥ 100, which were the top 5.8% high-connectivity proteins of the PIN. (**b**). Relative mean protein abundances. (**c**). Relative mean transcript abundances. Average values relative to the young cells at 7.8 h had been used. The cell ages were approximated to RLS using the dividing time of 100 min. The shade of each curve was calculated as the standard deviation from all proteins (genes) of the ratios of aging cells to the ground state, multiplied by the mean differences. The number of proteins (genes) used in the curves of each category had been listed.

The yeast genome has 5,191 verified, 725 uncharacterized, and 688 dubious ORFs (as of 9/17/2020) on the *Saccharomyces* Genome Database^34^. These ORFs encode ca. 1000 essential genes, with any one of which deleted the cell will not be viable^35–38^. We compared the abundances of 4 categories of proteins (genes) including essential hub, essential non-hub, nonessential hub, and nonessential non-hub proteins. The protein or transcript abundances were referenced to the ground state, the young cells with the age of 7.8 h, consistent with the PIPLs shown above. We used the node degree k ≥ 100 as a cutoff (which covered the top 5.8% high connectivity proteins) to classify the hub and non-hub proteins (Fig. 5a). The yeast life cycle length of 100 min was used to estimate the RLS^39^.

Consistent with the evaluation of the relative-quasi potentials (Methods and Materials), the node degrees were weighted to the protein or transcript abundance values in Fig. 5b,c. We observed that, at old age, the abundances of essential hub proteins increased considerably in the aging cells; however, the abundances of the essential non-hub proteins decreased (Fig. 5b). The opposite trends of the average essential protein abundances versus essential transcript abundances (Fig. 1) can be explained by the differences in the PIPL related to the essential proteins or transcripts. The differences between the average hub proteins and hub transcripts (Fig. 2) can also be explained here. We also listed the numbers of proteins (Fig. 5b) or transcripts (Fig. 5c) that were included in both the PIN the aging proteomics and transcriptomics data set. These trends of protein and/or transcript abundances may partly explain the basins and ridges observed in the protein interaction potential landscapes. Note that the absolute ages of the cells had been approximated to RLSs using an average division time of 100 min. This conversion may not be accurate, for example, the actual division time may increase during aging^40^, and the long estimated RLS in Fig. 5 (> 40) made it possible that some aging cells may have stopped division and stayed in the post-diauxic, quiescent phase^2^.

### The decline of the PPI dissociation constant may alter the landscapes

All PIPLs shown above were constructed using constant protein–protein interaction dissociation constants (*K*), i.e., the decay rate (d) is 0 for all PPIs. However, in reality, *K* might be affected or altered by many factors, such as post-translational modifications, protein aggregations, pH, ionic strengths, and other cofactors. It is difficult to measure the variations of *K* for each of the protein–protein interactions. We used an exponential decay model aiming to estimate the potential topological changes of the landscapes attributed to the variations of the dissociation constants, *K*.

**Figure 6:**
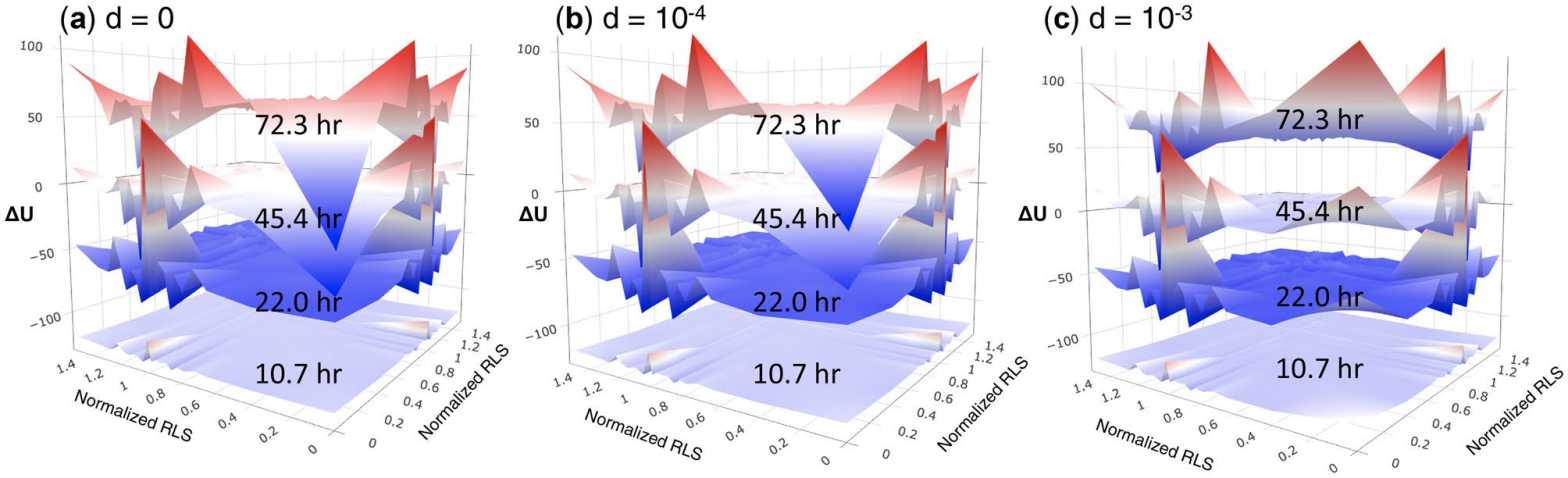
Proteome-based PIPL on RLS with dissociation constants exponentially decay. The decay rate (d, in the unit of 1/t) of the protein–protein interactions (PPIs) was chosen as **(a)** d = 0, from which *K*_*43*_ = *K*_*0*_, also see Fig. 1; **(b)** d = 10^–4^, from which *K*_*43*_ = *0.990 K*_*0*_; and **(c)** d = 10^–3^, from which *K*_*43*_ = *0.906 K*_*0*_, where the dissociation constants *K*_*43*_ (at the 43^th^ generation or ca. 71.7 h, close to the oldest cells in the analysis) was compared with the initial *K*_*0*_. The model with d = 0 **(a)** corresponds to constant dissociation constants in the models, which was applied to other PIPLs in the main text. However, there is a transition from d = 10^–4^ **(b)** to d = 10^–3^ **(c)**, in the latter PIPL the intra-essential interactions were located in a ridge for the old cells whereas in the former the intra-essential interactions were also in a basin for the old cells.

As shown in Fig. 6, in old yeast cells, the intra-essential interactions arose to a ridge (instead of falling to a basin) when *K* declines with a decay rate of 10^–3^—at this rate, *K*_*t*_ becomes half of *K*_*0*_ after 300 cell divisions. We noticed that the dissociation constant strongly affects the essential-essential interactions, partly contributed by their dense interactions. For example, in the proteome-based PIPL, the number of essential-essential interactions (n in Eq. 8) is 3465, ~ 10 times of that between essential proteins and any of the other 20 protein sets based on the quantiles of the normalized RLS.

The protein interaction potential landscapes on space of other parameters can also be constructed using our approach. As an example, the morphology parameters from the SCMD2 database^36^ had been used in the PIPL as shown in Figure S2 of the supplementary materials.

## Discussion

The roles of gene/protein networks in aging have been discussed extensively^11,12,14,41–44^, and the role of weakest links in aging was recognized^41–43^. A recent mathematical model suggested the role of essential genes in aging^14^. Loss of proteostasis is also a hallmark of aging^45^. The present work on the protein interaction potential landscape during aging suggest that essential hub genes and their interactions may be a critical factor of proteostasis and/or aging. Our results here pose an interesting hypothesis that essential hub genes might be one of the weakest links during aging, a question that could be addressed by future experimental studies.

The protein interaction potential landscapes of the budding yeast in the present work used the proteomic and transcriptomic data of aging cells mathematically unmixed from bulk cell culture with young daughter cells and dead cells^15^. We understand that potential biases may persist in the data set and hence in our interpretations. However, using these landscapes we showed that the aging dynamics could be visualized to identify potential aging factors, such as the variations caused by the essential hub proteins. Future studies using single-cell protein and/or transcript abundance data along aging could be combined with our approach to understanding the aging landscape. Moreover, the probability-based interaction landscapes can also be applied using other networks—such as the genetic interaction networks—in the understanding of the genotype–phenotype relationships^32,46^.

## Methods and materials

### Protein–protein interaction network

The empirical protein–protein interaction network (PIN) of *S. cerevisiae* was obtained from a recent version BioGRID v3.5.177, downloaded on Oct. 1, 2019^22^. Only the physical interactions related to proteins (excluding the self-interactions) were selected from this PIN, comprising 110,290 interactions among 5784 proteins.

### Network permutation-based association study (NetPAS)

The PIN used in the present work is regarded as a simple graph, i.e., an undirected graph with no self-loops or multi-interactions. We recently proposed the network permutation-based association study (NetPAS) approach to evaluate the interaction strength between different protein (gene) sets by comparing the original PIN and its random null models^17^. 22,188 null network models were built in a way such that all vertices were randomly reshuffled, yet the node degrees preserved as the original PIN. These network models were also termed the MS02 null models^17,30,47^.

### Replicative lifespan data

The replicative lifespans (RLSs) of 4698 single-gene deletion strains of *S. cerevisiae* under the YPD (2% glucose) culture had been reported^1^.

### Proteomics and transcriptomics data during aging

One set of proteomics and transcriptomics data of *S. cerevisiae* came from a recent report, which comprises the abundances of 1494 proteins and expression levels of 4904 genes collected at 12 different ages ranging from 7.8 to 72.3 h^15^. We used the young cells at 7.8 h as the reference (ground state) to evaluate the PIPLs of the older cells at the other 11 ages. The two data sets cover different genes (proteins), as shown in Figure S3 in the SM for a comparison.

### Protein turnover time

Two sets of in vivo protein turnover time or half-lives have been used. The CellSys2017 set^29^ comprised of 3160 proteins with 1341 in the aging proteomics and 2526 in the aging transcriptomics data sets. The CellRep2014 set^27^ included 3715 proteins with 1331 in the aging proteomics and 2988 in the aging transcriptomics data sets.

### Morphologies of single-gene deletion mutants

We also generated the PIPLs on dimensions of morphological parameters and the RLS of the yeast single-gene deletion mutations. See in the supplementary materials. The morphology data were taken from the SCMD2 (Saccharomyces cerevisiae morphological database 2)^36^. Specifically, two morphologies related to a recent morphological landscape of yeast replicative aging had been analyzed in the protein interaction landscapes^10,48^, including the cell size ratio (C118_C) and bud axis ratio (C114_C) at the late stages. Although the definition of the axis ratio in SCMD2 was different than that used in the morphological landscape, it could provide a rough identification of the roundness of the cell shapes.

### Essential genes of *S. cerevisiae*

The essential gene set of *S. cerevisiae* were taken from the SCMD2^36^ that comprises 1112 genes, 1033 of which have interactions recorded in the PIN.

### Selection of gene groups

We used quantiles to catalog the gene groups in the PIPLs. The yeast PIN covered 3939 genes with the RLS records of the single-gene deletion mutations. The normalized RLS refers to the RLS ratios between the mutant and the control wide type strain and had been adopted in the landscapes. Among the genes covered by the PIN, 926 non-essential and 403 essential genes had the age-dependent protein abundance records, and 2991 non-essential and 882 essential genes had the age-dependent transcript records, respectively. In both the age-dependent protein abundance and transcript level sets, the non-essential genes were evenly classified into 20 groups based on the quantiles of the normalized RLSs, and the essential genes were classified into one group with the normalized RLS of 0. For the protein node degrees, half-lives, and morphology data sets, all genes were classified into 20 evenly distributed quantiles based on the respective measures. The same classification approach for the RLS groups was applied to the global protein interaction landscape, and all genes included in the PIN and with normalized RLS data had been used.

### Quasi-potentials of age-dependent protein interaction potential landscape

Aging is a dynamic process reflected in the variations of both protein abundances and transcript levels along the aging process. It had been suggested that a configuration of the cell state could be formalized as a vector

1 $${\mathbf{S}}_{{\mathbf{t}}} = \, \left[ {{\text{x}}_{{1}} ,{\text{ x}}_{{2}} , \, \ldots ,{\text{ x}}_{{\text{N}}} } \right]_{{\text{t}}} ,$$

where N is the total number of genes and x_i_ is the expression level of the i-th gene^49^—we suggest x_i_ could also be the protein abundance of the i-th protein. The vector **S**_**t**_ continuously changes during the aging process.

The interaction between a pair of proteins x and y can be written as

2 $$x + y\overset{K} \to xy$$

we can approximately apply the law of mass action to this process in which the concentration [xy] is

3 $$\left[ {xy} \right]_{t} = { }K\left[ x \right]_{t} \left[ y \right]_{t} = { }p_{t}$$

where *K* is the dissociation constant and [x]_t_ and [y]_t_ is the concentration of free x and y at time t. *K* may variate along aging, e.g., via an exponential model

4 $$K_{t} = K_{0} 10^{ - dt}$$

here, *d* is the decay rate, and t is time (age), and *K*_t_ and *K*_0_ are the dissociation constants at age t and 0, respectively. The decay rate may be affected by posttranslational modifications, pH and ionic strength variations during aging, and other factors. The dissociation constant *K* remains unchanged during aging if d is 0; *K* exponentially decays or increases if d > 0 or d < 0, respectively. Under this condition and by using the young cell (ground state) at time 0 as the reference state or ground state, we could get:

5 $$p_{t} /p_{0} = \left[ {xy} \right]_{t} /\left[ {xy} \right]_{0} = \left( {\left[ x \right]_{t} \left[ y \right]_{t} } \right)/\left( {\left[ x \right]_{0} \left[ y \right]_{0} } \right) \times 10^{ - dt} .$$

Note that Eq. (5) does not contain *K*_0_, which was canceled by comparing with the ground state. Therefore, using a reference state at time 0 (i.e., the ground state), the interactions between different protein pairs could be treated equally, if the variation rates of the dissociation constants are the same.

Besides the above approximation, we used a quasi-potential, the negative logarithm of the probabilities, to describe the barriers that the cells must overcome for the transition from one attractor state to the other^49,50^. The quasi-potential^49^ can be calculated from the probabilities using

6 $$U = - {\text{log}}p .$$

Again, because all potentials here are relative to the ground state, the relative quasi potential U_t_ using U_0_ as a reference can be calculated as

7 $$\Delta U_{t} = U_{t} - U_{0} = - \log \left( {\frac{{\left[ {xy} \right]_{t} }}{{\left[ {xy} \right]_{0} }}} \right) + dt = { } - \log \left( {\frac{{\left[ x \right]_{t} }}{{\left[ x \right]_{0} }}} \right) - \log \left( {\frac{{\left[ y \right]_{t} }}{{\left[ y \right]_{0} }}} \right) + dt .$$

Equation (7) provides a convenient way to estimate the relative quasi potentials at time t, compared to the young cell ground state.

In this work, the relative quasi potential of interactions between two categories of proteins (or genes) is evaluated. The probability is not additive as we cannot sum up the probabilities of different interactions. However, the logarithm function and hence the quasi potential U is additive. Therefore, it is straightforward to calculate the relative quasi potential of interactions between two sets of genes X and Y based on Eq. (7). For instance, assuming that there are n interactions between X and Y recorded as (x_i_, y_i_), *i* = 1, 2, …, n, the relative quasi-potential from all interactions between X and Y would be

8 $${\Delta }U_{t} = - \mathop \sum \limits_{i = 1}^{n} \log \left( {\frac{{\left[ {x_{i} } \right]_{t} }}{{\left[ {x_{i} } \right]_{0} }}} \right) - \mathop \sum \limits_{i = 1}^{n} \log \left( {\frac{{\left[ {y_{i} } \right]_{t} }}{{\left[ {y_{i} } \right]_{0} }}} \right) + ndt.$$

The base of the logarithm function (Eqs. 6–8) is 10 in present work. It should be noted that in present work all quasi-potentials of every single interaction were relative to the ground state and could therefore bear any real values. The unit of the protein abundances or mRNA levels (such as FPKM) were canceled out and did not affect the relative quasi-potentials in Eqs. (5) -(8). In the landscapes of this work, we observed high relative quasi-potentials especially when the interactions between two categories of proteins were dense. However, a cutoff of ± 50 was applied in the quasi-potentials of all contour maps because it could capture the indispensable information yet provides a relatively good comparison among different landscapes. The variations of the dissociation constant cannot be justified in this work, however, we showed that significant changes of the landscape could be induced by a relatively slow decay rate (*d*) for the dissociation constants of the PPIs (Fig. 6).

In summary, in the present work we apply a simple statistical approach to approximate the interaction probabilities of protein groups using either the protein abundances or the gene expression levels. Then quasi-potentials based on these probabilities were visualized as landscapes, which may infer the variations of interaction patterns caused by the aging process.

### Quasi-potentials of proteome-wide protein-interaction potential landscape

Here we calculated the protein-interaction quasi potentials based on the probability (*p*) for more interactions between certain categories of genes in the PIN than in the null models. The NetPAS method^17^ was used to calculate the *p*-values using 22,188 null models. The protein-interaction quasi potential can also be calculated using Eq. (6). The base of the logarithm function may vary for the conceptual barriers in Eq. (6). As 22,188 null models were used, the occurring *p* = 0 cases could be interpreted as *p* < 1/22,188, which was converted to *p* = 1/22,189 in practice to avoid the infinite log0 values. The resulted quasi-potentials were then normalized to [0,1] for the landscape.

## Supplementary Information

Supplementary Figures.
