# Supplementary material for: Protein interaction potential landscapes for yeast replicative aging
Source: Sci Rep. 2021 Mar 30;11:7143. doi: 10.1038/s41598-021-86415-8 (PMC8010020; doi:10.1038/s41598-021-86415-8)
Supplement: Supplementary file 1 — Supplementary Figures. [file 41598_2021_86415_MOESM1_ESM.docx]

***Supplementary Materials for***

Protein interaction potential landscapes for yeast replicative aging

Hao-Bo Guo^1,2,*^, Mehran Ghafari^1^, Weiwei Dang^4^ , Hong Qin^1,2,3,*^

^1^Department of Computer Science and Engineering

^2^SimCenter

^3^Department of Biology, Geology and Environmental Science

The University of Tennessee at Chattanooga, Chattanooga, Tennessee 37405, USA

^4^Huffington Center on Aging, Baylor College of Medicine, Houston, Texas 77030, USA

*Correspondence: guohaobo@gmail.com (HG) and hong-qin@utc.edu (HQ)

The supplementary materials include:

**Figure S1**. High interaction density was found between essential and hub proteins.

**Figure S2**. The PIPLs on dimensions of RLS and morphologies.

**Figure S3**. A Venn diagram for the genes covered by the proteomics and transcriptomics data sets.

**Figure S1**. High interaction density was found between essential and hub proteins. The average interaction density calculated on dimensions of node degree and RLS ratios (normalized RLSs) is shown in (**a**) landscape and (**b**) contour map. The interaction density between two sets of nodes is defined as the total number of interactions divided by the product of the sizes of both sets.

**Figure S2**. The PIPLs on dimensions of RLS and morphologies. The PIPLs were based on protein abundances (left) or gene expressions (right). The morphologies include (**a**) The daughter-to-mother cell size ratios and (**b**) the daughter-to-mother cell size ratios. Both morphologies were measured at the late stage of the daughter cell growth, details could be found in the SCMD2^32^. The landscapes at different ages have been vertically shifted for better visualization. The color bar shows the relative quasi-potentials in the contour maps. A relative color scheme (blue for low and red for high) is applied in landscapes.

A recent work proposed a morphological landscape of yeast aging on which the mother cells took two distinguishable aging paths: in the first path the mother cells produced enlarged and elongated daughter cells, and in the second path the mother cells produced small and round daughter cells^45^. The mother cells went through the first path had longer RLSs than the mother cells underwent the second path. Two morphologies were used in this landscape to infer the aging paths including the daughter-to-mother size ratios for daughter cell size and the axis ratios of the daughter cells for daughter cell roundness, both at the late stages of cell growths^10,45^. Here, we combined the RLSs and took advantage of the Saccharomyces Cerevisiae Morphological Database (SCMD2) which recorded 501 morphologies of different yeast single-gene deletion mutants^32^. Figure S1 showed both the protein abundance-based and gene expression-based PIPLs of RLS versus morphologies obtained from the SCMD2 including the daughter-to-mother size ratios (C118_C) and daughter axis ratios (C114_C) at the late stages of cell growth from the SCMD, both had been used in the construction of the morphological landscape^45^.

Obvious differences between the landscapes based on protein abundances and those based on gene expressions could be found, similar to the landscapes shown above. It should be noted that the morphologies selected from SCMD2 were based on high-resolution images of single-gene deletion mutations^32^ such that were different than those in the recent morphological landscape^10,45^. Moreover, the morphological differences in the early and late stages of the daughter cell growth had also been considered ^32^. We did observed differences of the absolute values of the morphologies of cells at the early growth stage versus those at late growth stage, however, the orders of these genes were not necessarily conserved. These landscapes could therefore be useful to interpret the general trends related to the morphologies. For example, at an old age, the genes related to small daughter cells (Figure S1) seem to have larger fluctuations in the interactions with other proteins.

The landscapes from the present work described the protein-interaction probabilities (or relative quasi-potentials) of single-gene deletion mutants, and they did not represent cell states. Therefore, these landscapes did not predict how the morphological variations affected the RLS of mother cells as the cell-state landscapes^10,45^. Instead, the landscapes of present work suggest that the PINs in the old cells had been altered by aging. To rejuvenate to the young cell state, the old yeast cells needed to redistribute the interactions in the PIN, especially from essential genes, to flatten the above age-dependent landscapes.


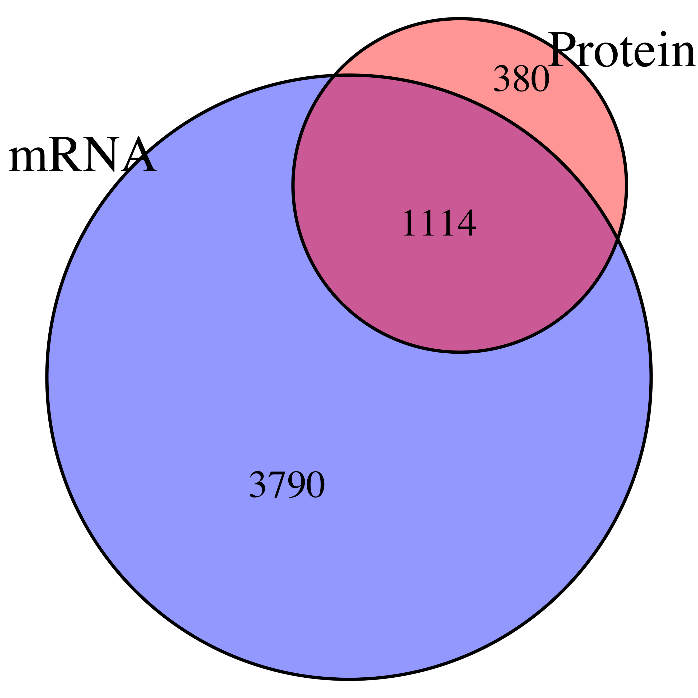


**Figure S3**. A Venn diagram for the genes covered by the proteomics (red) and transcriptomics (blue) data sets.

The aging-dependent proteome and transcriptome sets cover different genes, yet they also show significant overlaps, comprising 74.5% of proteins of the proteomics data set, see Figure S3.
